# Supplementary material for: Prolonged Subculturing of Aspergillus fumigatus on Galleria Extract Agar Results in Altered Virulence and Sensitivity to Antifungal Agents
Source: Cells. 2023 Mar 31;12(7):1065. doi: 10.3390/cells12071065 (PMC10093746; doi:10.3390/cells12071065)
Supplement: Supplementary file 1 [file cells-12-01065-s001.zip › cells-2266326-supplementary.pdf]

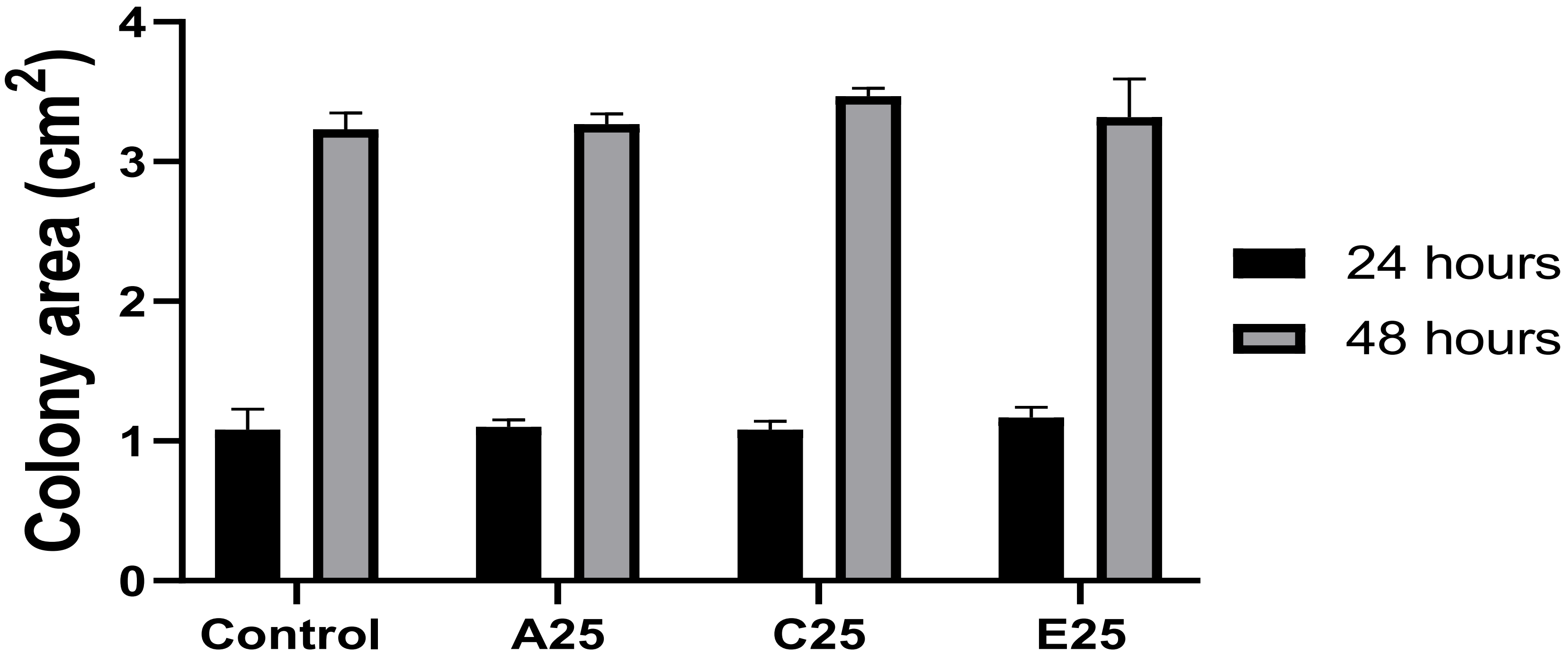

Figure S1. Colony area of passaged and control *A. fumigatus* on Malt extract agar over 48 hours. No significant alteration was observed in the growth rate of the strains.

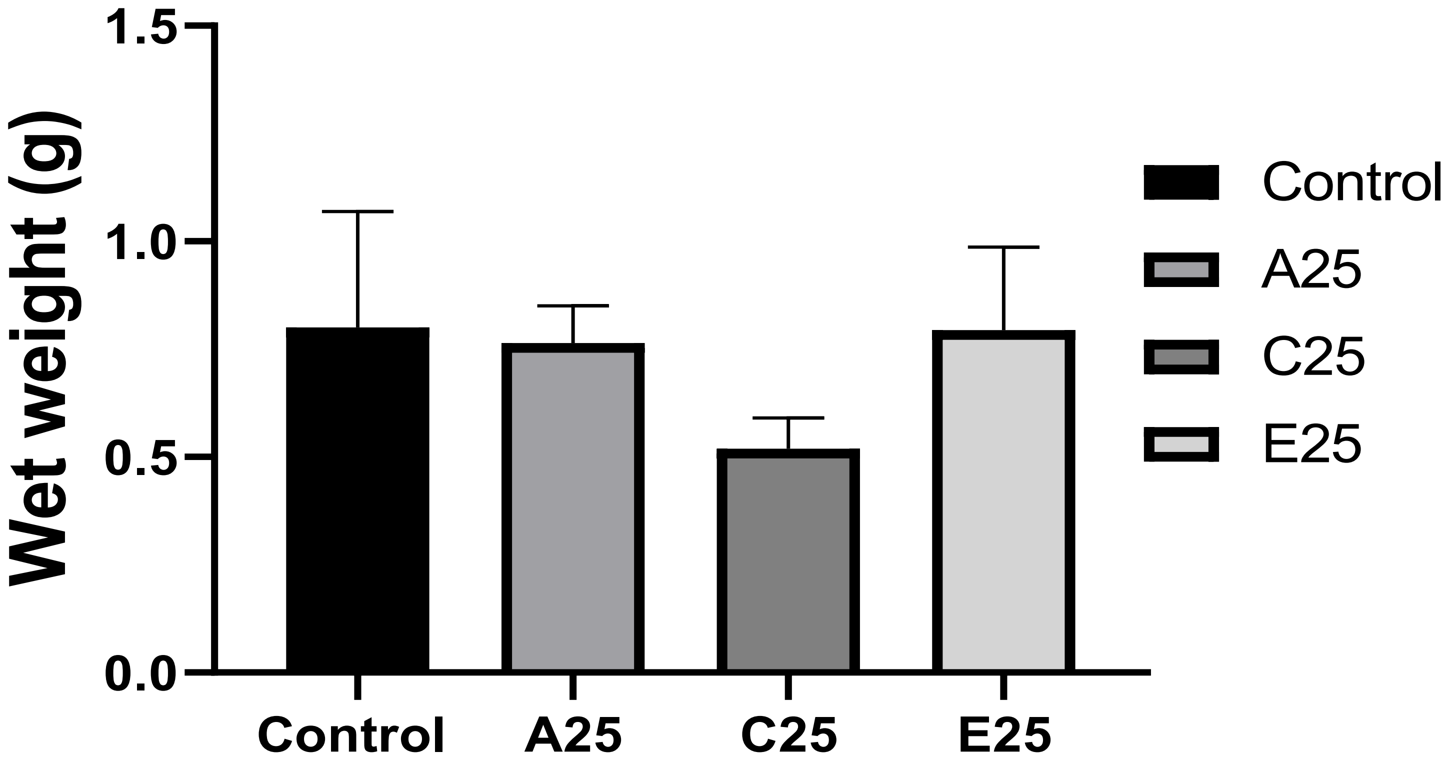

Figure S2. Mycelial wet weight of passaged and control *A. fumigatus* grown in Czapex Dox broth for 72 hours
No significant alteration was observed in the wet weights of the strains.

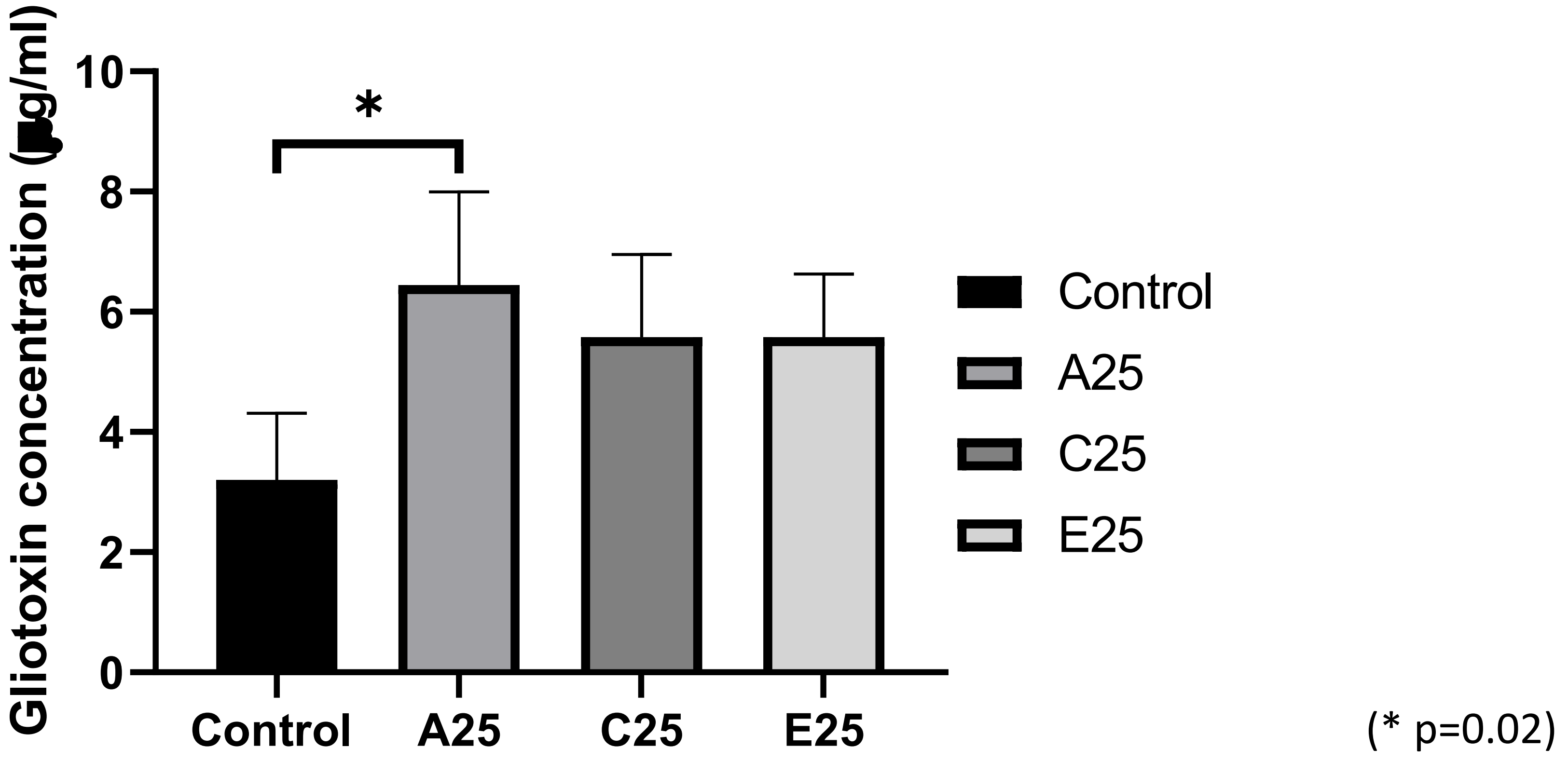

Figure S3. The gliotoxin concentration in culture filtrates of passaged and control *A. fumigatus* grown in Czapex Dox broth for 72 hours.

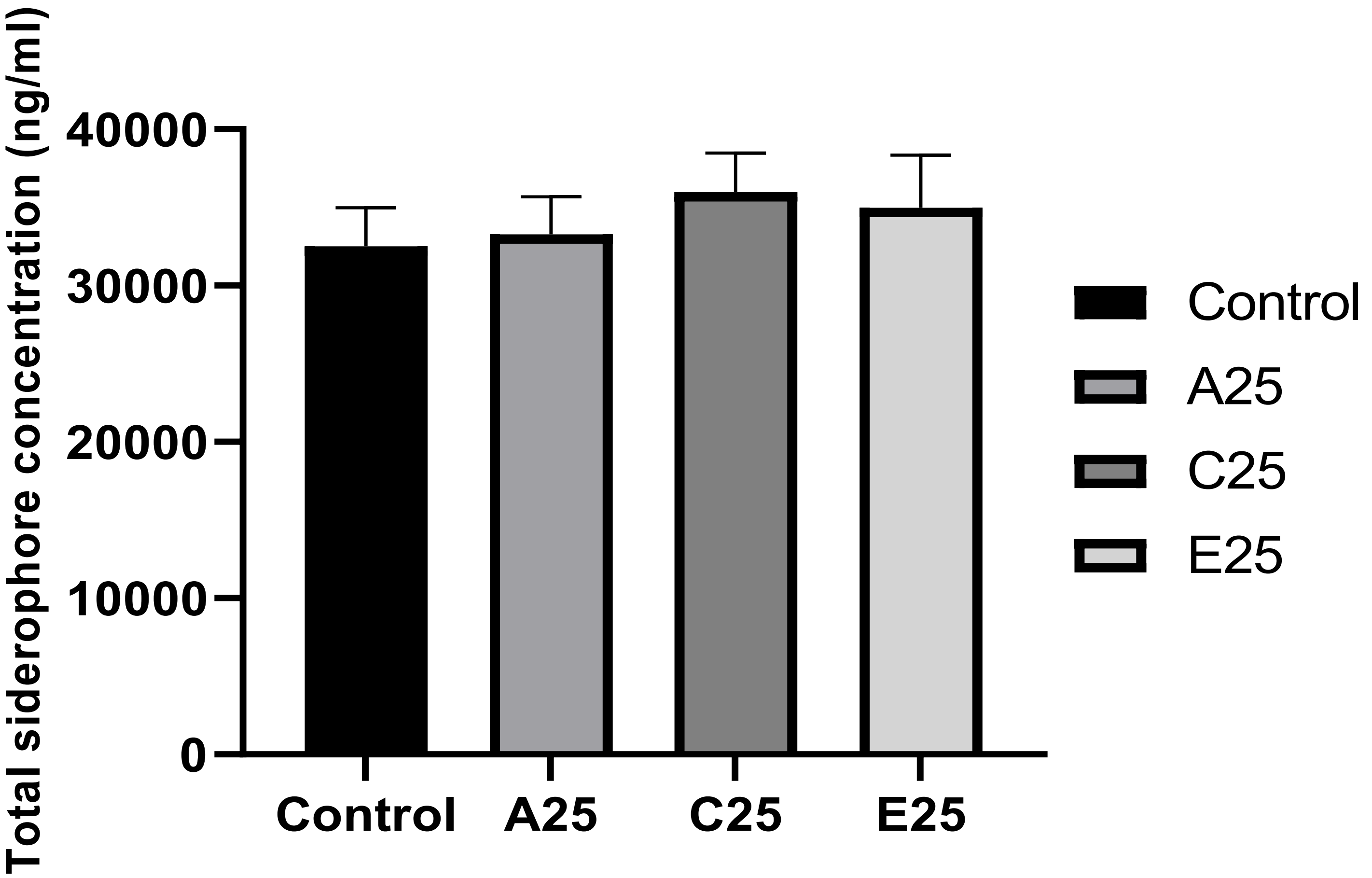

Figure S4. The concentration of siderophore in culture filtrate of passaged and control *A. fumigatus* grown in Czapex Dox broth for 72 hours.

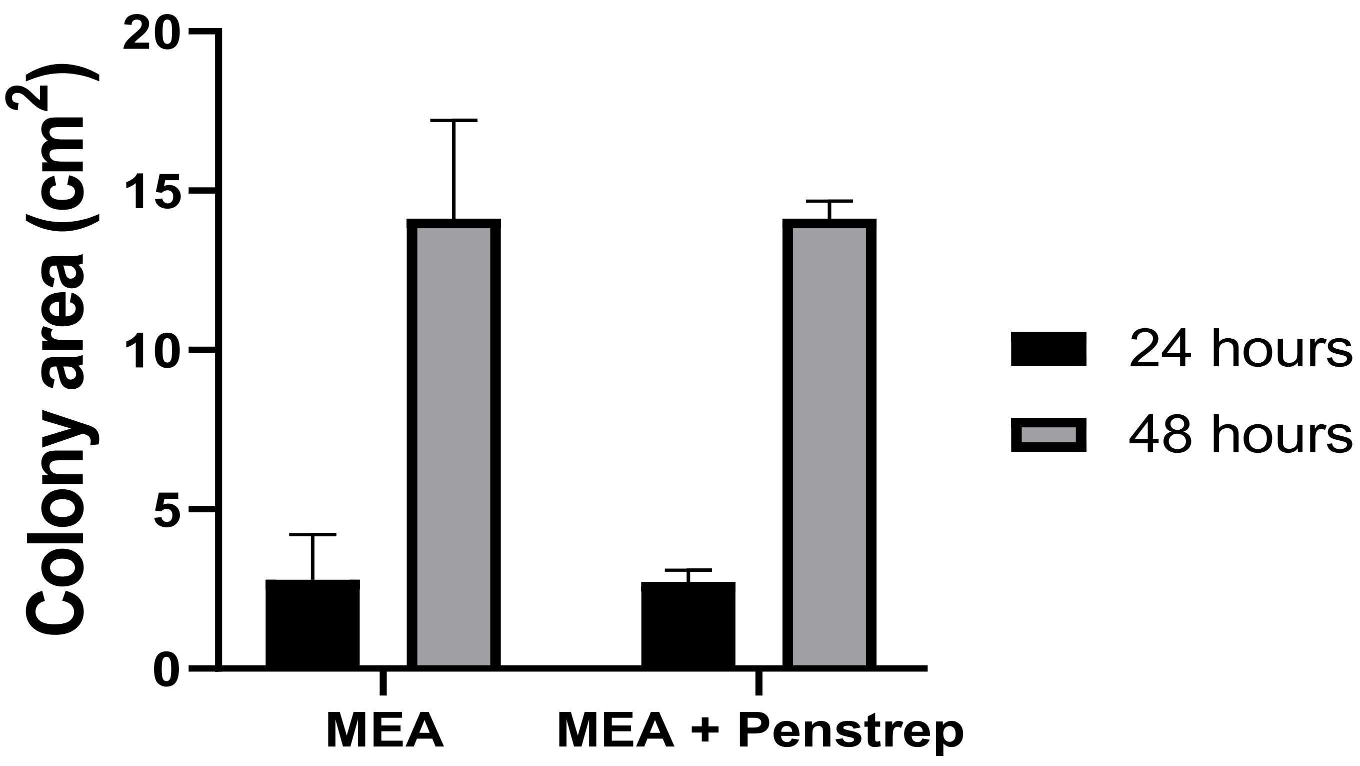

Figure S5. Radial growth of control *A. fumigatus* and *A. fumigatus* grown on MEA supplemented with 0.1% (v/v) penstrep, for 48 hours.

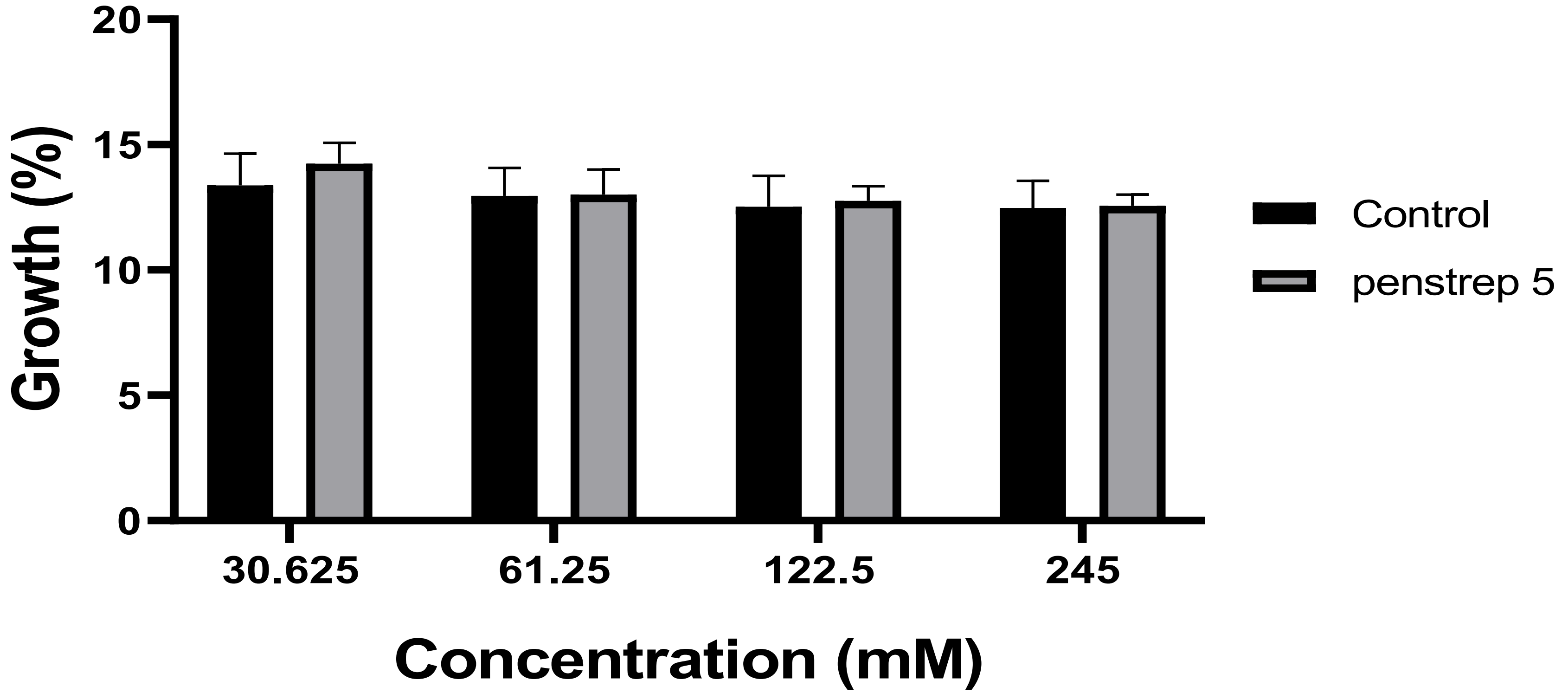

Figure S6. Response of control and penstrep-passaged *A. fumigatus* to hydrogen peroxide. *A. fumigatus* grown in the presence of 0.1% (v/v) penstrep for five passages demonstrated no significant alteration in response to hydrogen peroxide.

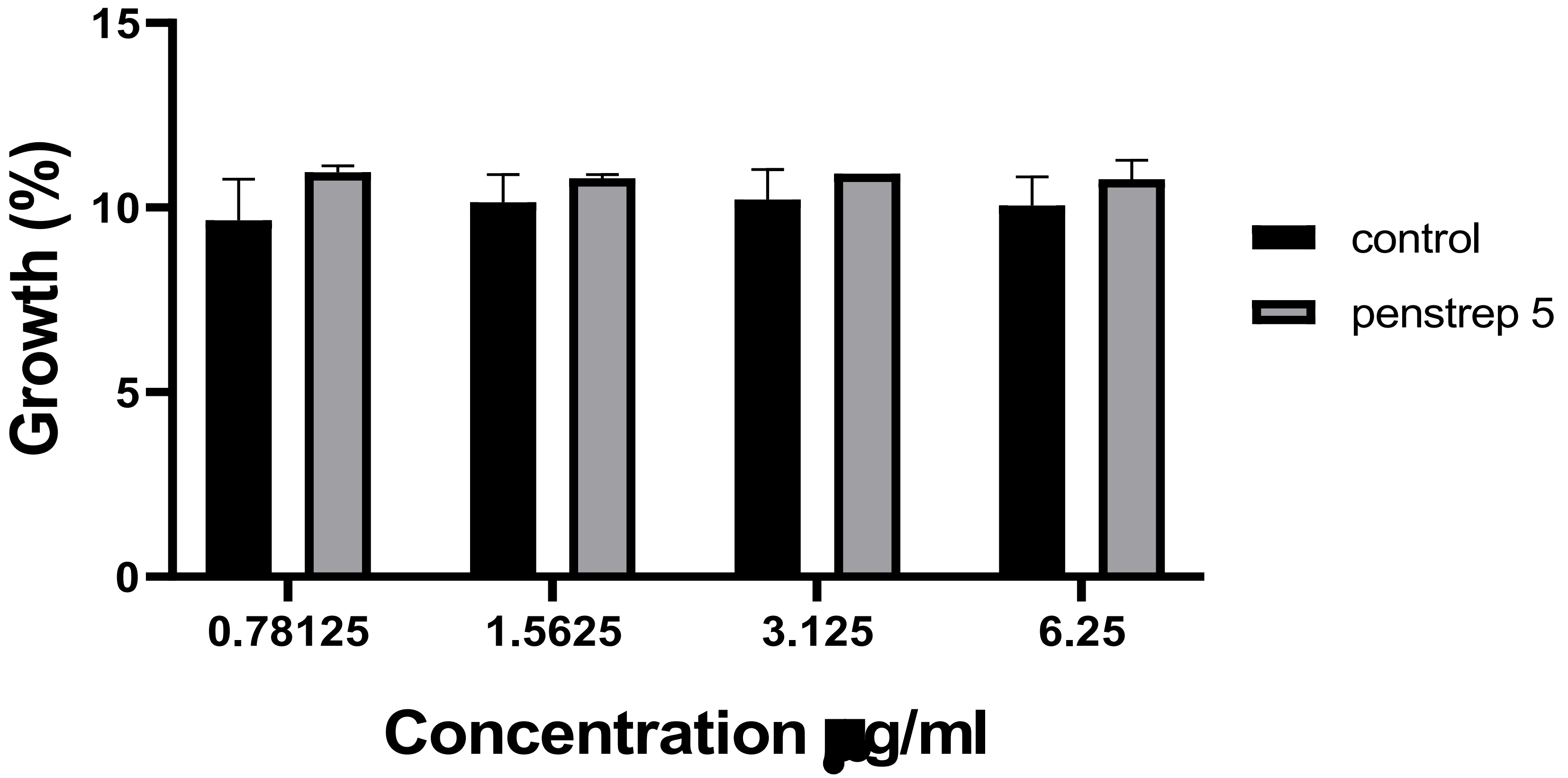

Figure S7. Response of control and penstrep-passaged *A. fumigatus* to Amphotericin B. *A. fumigatus* grown in the presence of 0.1% (v/v) penstrep for five passages demonstrated no significant alteration in response to Amphotericin B.

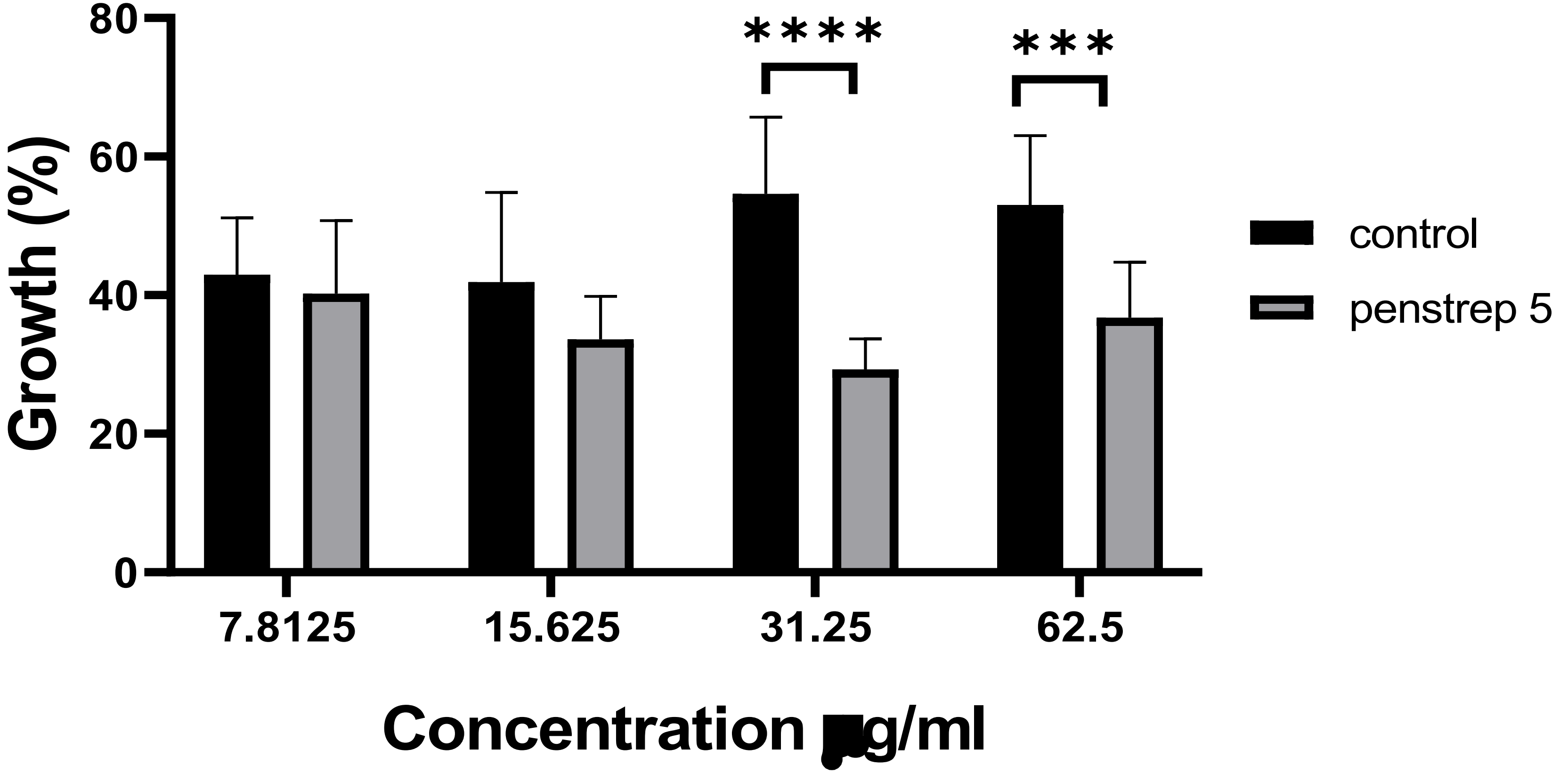

Figure S8. Response of control and penstrep-passaged *A. fumigatus* to itraconazole. *A. fumigatus* grown in the presence of 0.1% (v/v) penstrep for five passages demonstrated increased susceptibility to itraconazole at 31.25 and 62.5 μg/ml. (*** p = 0.001, ****p = 0.0001)
